# Bone morphogenetic protein signalling in pulmonary arterial hypertension: revisiting the BMPRII connection

**DOI:** 10.1042/BST20231547

**Published:** 2024-05-08

**Authors:** Wei Li, Kate Quigley

**Affiliations:** VPD Heart and Lung Research Institute, Department of Medicine, University of Cambridge School of Clinical Medicine, Cambridge CB2 0BB, U.K.

**Keywords:** BMP signalling, cardiovascular disease, extracellular regulation of signalling, molecular mechanisms, pulmonary arterial hypertension, therapeutics

## Abstract

Pulmonary arterial hypertension (PAH) is a rare and life-threatening vascular disorder, characterised by abnormal remodelling of the pulmonary vessels and elevated pulmonary artery pressure, leading to right ventricular hypertrophy and right-sided heart failure. The importance of bone morphogenetic protein (BMP) signalling in the pathogenesis of PAH is demonstrated by human genetic studies. Many PAH risk genes are involved in the BMP signalling pathway and are highly expressed or preferentially act on vascular endothelial cells*.* Endothelial dysfunction is recognised as an initial trigger for PAH, and endothelial BMP signalling plays a crucial role in the maintenance of endothelial integrity. *BMPR2* is the most prevalent PAH gene, found in over 80% of heritable cases. As BMPRII protein is the major type II receptor for a large family of BMP ligands and expressed ubiquitously in many tissues, dysregulated BMP signalling in other cells may also contribute to PAH pathobiology. Sotatercept, which contains the extracellular domain of another transforming growth factor-β family type II receptor ActRIIA fused to immunoglobin Fc domain, was recently approved by the FDA as a treatment for PAH. Neither its target cells nor its mechanism of action is fully understood. This review will revisit BMPRII function and its extracellular regulation, summarise how dysregulated BMP signalling in endothelial cells and smooth muscle cells may contribute to PAH pathogenesis, and discuss how novel therapeutics targeting the extracellular regulation of BMP signalling, such as BMP9 and Sotatercept, can be related to restoring BMPRII function.

## Pulmonary arterial hypertension and bone morphogenetic protein signalling

Pulmonary arterial hypertension (PAH) is a rare but debilitating condition with a high mortality rate, affecting 15–26 people per million of the population in western countries [1,2]. The pathology is characterised by the abnormal muscularisation of pre-capillary pulmonary arteries, formation of concentric and plexiform lesions and narrowing of the pulmonary vascular lumen, resulting in an increase in pulmonary vascular resistance, elevated pulmonary artery pressure, right ventricle hypertrophy, and progressive right heart failure [3]. The FDA approved PAH therapies prior to 2024 target three pathways that predominantly affect vascular tone: endothelin 1, nitric oxide and prostacyclin. Although these therapies have improved exercise capacity and delayed clinical worsening time, they do not provide a cure for most patients and survival at three years post-diagnosis remains unacceptably low [4]. Therapies directly targeting the underlying disease pathophysiology are urgently needed.

Genetic studies suggest that PAH can be caused by pathogenic germline mutations. The most prevalent disease gene is *BMPR2* (bone morphogenetic protein (BMP) receptor 2) [5,6], encoding the type II receptor for the large family of BMP ligands. *BMPR2* mutations are found in over 80% of familial cases and ∼17% of idiopathic PAH (IPAH) patients [7–9]. Among the 12 validated PAH genes that have been recognised by the International Consortium for Genetic Studies in PAH [10], many are related to BMP signalling. Apart from *BMPR2*, *ACVRL1* encodes BMP type I receptor activin receptor-like kinase 1 (ALK1), *ENG* encodes co-receptor endoglin, *GDF2* encodes ligand BMP9, and *SMAD9* is a component of BMP signalling machinery [6,11]. Of note, mutations in PAH genes are predisposing factors with incomplete penetrance. *BMPR2* variants penetrance is estimated to be 42% for heterozygous women and 14% for heterozygous men [6,12].

## Introduction to BMP signalling

BMPs are members of the transforming growth factor-β (TGF-β) superfamily. These ligands are mostly homodimers, initiating cellular signalling by forming a complex with cell surface receptors comprising two copies of a type I receptor and two copies of a type II receptor. Both types of receptors are serine/threonine kinases. After formation of the signalling complex, the constitutively active type II receptor will phosphorylate and activate the type I receptor, which will in turn phosphorylate the receptor-regulated SMADs (R-SMADs, including SMAD1, SMAD5, SMAD9, SMAD2 and SMAD3). The phosphorylated R-SMADs then form a complex with the common mediator SMAD, SMAD4, and translocate to the nucleus to regulate gene expression. Typically, BMP signals are mediated by SMAD1, SMAD5 and SMAD9, whereas signals from TGF-βs, Nodal, activins and some growth and differentiation factors (GDFs) are mediated by SMAD2 and SMAD3. Signalling from the TGF-β family ligands can also involve non-SMAD pathways such as p38, ERK1/2 and PI3K which impact on cell proliferation, apoptosis and migration [13]. A more comprehensive review on TGF-β family signalling has been published recently [14].

**Figure 1.**
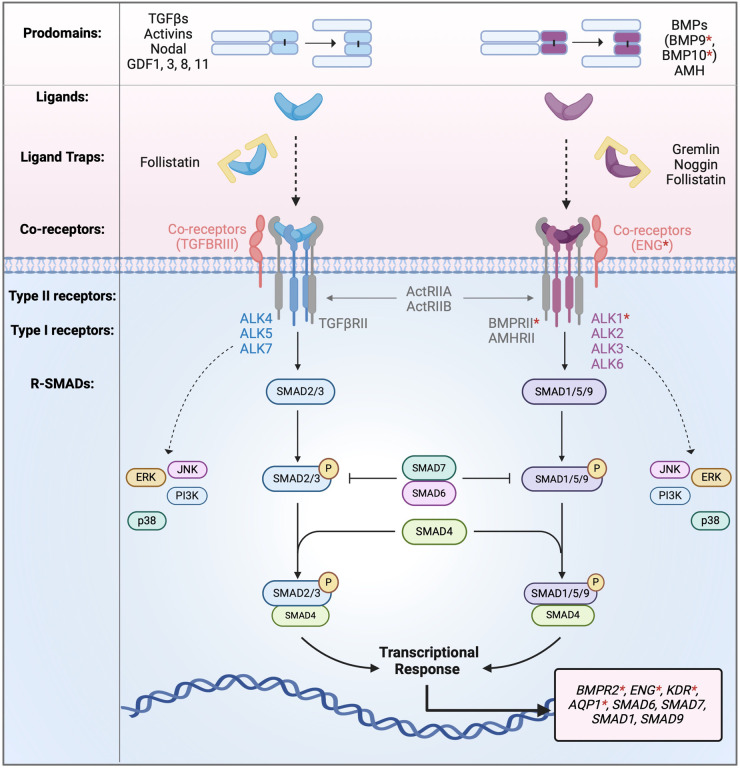
Introduction to the highly dynamic TGF-β and BMP signalling. TGF-β family signalling is regulated at multiple levels including: (1) TGF-β family ligands are synthesised and secreted as the prodomain bound forms; (2) there are a large number of ligands signalling through a limited number of type I and type II receptor pairs with a high degree of promiscuity in ligand-receptor interaction; (3) extracellular ligand traps can bind ligands and prevent them from binding to the receptors; (4) co-receptors can modify ligand-receptor interactions through direct protein-protein interactions; (5) TGF-β and BMP signalling can regulate the gene expression of components on the signalling pathways, such as *SMAD9*, *BMPR2* and *ENG*, or inhibitors of the signalling pathways, such as *SMAD6* and *SMAD7*. Genes (and encoded proteins) that are mutated in PAH are highlighted by *.

TGF-β family ligands are encoded by a total of 33 genes in humans, yet there are only 7 type I and 5 type II receptors mediating their signals, hence there is a high degree of promiscuity in ligand-receptor interactions. One BMP ligand can signal via different type I and type II receptor pairs, and the same type I and type II receptor pair can mediate signals from different ligands. In addition to ligand-receptor interaction, each ligand is synthesised and secreted as a prodomain bound complex; the prodomain may modify ligand bioactivity or localisation [15,16]. The extracellular regulation of BMP signalling also involves ligand traps (inhibitors) which limit ligand availability to the receptors, and cell surface co-receptors (also called type III receptors) which can modify ligand-receptor interactions [11]. Therefore, the overall signalling outcome is highly context dependent and determined by local concentrations of different ligands, ligand traps, and cell surface receptors and co-receptors [17] (Figure 1).

TGF-β and BMP signalling are also regulated at intracellular levels. Inhibitory SMADs, such as SMAD6 and SMAD7, are target genes of many ligands and can directly inhibit BMP and TGF-β signalling. Some BMPs also regulate the expression of their own receptors and co-receptors, for example, BMP9 and BMP10 induce the expression of *BMPR2*, *ENG*, *SMAD9*, and suppress *SMAD1* expression in endothelial cells (Figure 1). Such intracellular regulation provides feedback loops, ensuring a highly dynamic yet tightly controlled BMP signalling outcome.

With such complex regulation mechanisms, it is essential to establish how mutations in different genes lead to the dysregulated BMP signalling and contribute to the pathobiology of PAH. In addition, it is essential to understand how *BMPR2* mutations might affect signals from different TGF-β family ligands and in different cell types, which could contribute to the initiation or exacerbation of PAH.

## Dysregulated endothelial BMP signalling in the pathogenesis of PAH

Genetic findings strongly support the crucial role of dysregulated endothelial BMP signalling in the initiation of PAH. Several genes that are mutated in PAH encode proteins that are part of BMP signalling complex and highly expressed in vascular endothelial cells, such as *BMPR2*, *ACVRL1* and *ENG*. Importantly, *ACVRL1* is almost exclusively expressed in endothelial cells, mediating signals from two specific ligands, BMP9 and BMP10. Mutations in both *GDF2* (encoding BMP9) and *BMP10* have been identified in PAH patients. The clinical phenotypes of PAH patients with *GDF2* and *BMP10* mutations have been characterised in a recent report [18].

Endothelial dysfunction, which includes endothelial cell apoptosis, compromised barrier function, and altered vasoactive mediator release, etc, plays a central role in the initiation of PAH [19]. Reduced or loss of endothelial *BMPR2* expression leads to endothelial dysfunction. *In vitro*, a reduction or loss of *BMPR2* in human pulmonary vascular endothelial cells induces mitochondrial dysfunction and promotes a pro-inflammatory and pro-apoptotic state [20], causes endothelial-to-mesenchymal transition [21], and induces apoptosis [22,23] and excess permeability [22,24]. BMPRII deficiency impairs apoptosis via the BMPRII-ALK1-BclX (B-cell lymphoma X)-mediated pathway and the Bcl-xL isoform could be a potential biomarker for PAH [25]. *In vivo*, loss of *Bmpr2* causes increased lung vascular permeability [22]. Conditional deletion of *Bmpr2* in the pulmonary endothelium [26] or knocking-in human mutation R899X into *Bmpr2* gene [22] predisposes mice to PAH. Circulating BMP ligands, mostly BMP9 and BMP10, act constitutively and potently on vascular endothelial cells [27,28], inducing *BMPR2* expression [29] and have a plethora of endothelial protective functions, including anti-apoptosis, anti-migration, anti-proliferation, and anti-angiogenesis [22]. Administration of BMP9 neutralising antibody in adult mice leads to excess permeability in lung vasculature [30].

## Crystal structures of the BMPRII signalling complex reveal the highly dynamic interaction between BMP10 and BMPRII

Although *BMPR2* mutations in PAH were first published in 2000, structural insights into how BMPRII interacts with a BMP ligand were only reported in 2022 [31]. Crystal structures of BMPRII extracellular domain (ECD) in complex with BMP10, and in complex with both BMP10 and ALK1 ECD, revealed an unprecedented degree of plasticity in the BMPRII:BMP10 interaction [31]. This suggests that stabilising the interaction between BMPRII and BMP10 requires high concentrations of BMPRII, and that under normal physiological conditions, BMPRII-dependent signalling is most active in tissues with the highest *BMPR2* expression. As lung vascular endothelial cells have the highest expression of *BMPR2*, along with the high expression of BMP10 in the right atrium, together they partly explain why *BMPR2* mutations which cause haploinsufficiency will have the most impact on lung vasculature [31].

## *BMPR2* mutations also cause dysregulated BMP signalling in pulmonary smooth muscle cells

BMPRII is the type II receptor for all BMPs, and ubiquitously expressed in many cell types. Germline mutations in *BMPR2* also affect its expression in non-endothelial cells. In pulmonary artery smooth muscle cells (PASMCs) isolated from PAH patients harbouring *BMPR2* mutations, BMP4-induced SMAD1 phosphorylation and *ID1* gene expression were reduced [32,33]. The growth suppressive response to BMP4 was lost in proximal PASMCs harbouring *BMPR2* mutations [32].

BMP6 and BMP7 also signal in PASMCs. In one study, it was shown that the induction of *ID1* and *ID3* gene expression by BMP6 treatment was reduced in *BMPR2* mutant PASMCs [33], In another report, the result was more complicated [34]. In this latter study using mouse *Bmpr2*^−*/*−^ and *Bmpr2^+/^*^−^ PASMCs, it was shown that while BMP7 signalling was reduced in *Bmpr2^+/^*^−^ PASMCs, there was a gain of BMP6 and BMP7 signalling in *Bmpr2*^−*/*−^ PASMCs, even when BMP2 and BMP4 signalling remained reduced upon complete knockout of *Bmpr2*. This suggests that after a threshold change of cell surface BMPRII to somewhere below 50%, BMP6 and BMP7 gain of signal appears. More interestingly, in these *Bmpr2*^−*/*−^ PASMCs, ActRIIA took over to mediate BMP4 and BMP6 signalling, and the type I receptor preference changed. ActRIIA can pair up with both ALK2 and ALK3 to mediate BMP4 signalling in *Bmpr2*^−*/*−^ cells, whereas BMP6 (or BMP7) employs ALK2 only when BMPRII is absent [34]. Such gain of BMP6 signalling was also observed in PASMCs when BMPRII was shed from cell surface and *BMPR2* mRNA levels were reduced by more than 50% upon tumour necrosis factor-α (TNF-α) treatment [35].

## Altered inflammatory response exacerbates loss of BMPRII function and contributes to PAH pathogenesis

The penetrance of gene mutations causing PAH is low, and a second hit is often present in the pathogenesis of PAH. Inflammation is widely accepted as a major second hit for PAH, and multiple studies have shown inflammation further contributes to dysregulated BMP signalling in pulmonary vascular cells. BMPRII deficiency promotes an exaggerated inflammatory response in human and mouse SMCs, producing higher levels of IL-6 and IL-8 after LPS-stimulation compared with controls [36]. TNF-α causes reduced mRNA expression of *BMPR2* in both human pulmonary artery endothelial cells (hPAECs) and PASMCs [35], thus exacerbating the loss of BMPRII protein function in these vascular cells. *BMPR2* deficiency in PASMCs conferred insensitivity to TGF-β induced growth inhibition, and this process is associated with enhanced IL-6 and IL-8 induction by TGF-β [37]. IL1-β drives an exaggerated inflammatory response when *BMPR2* is deficient in PASMCs [38]. On the endothelial cell front, loss of *BMPR2* leads to increased permeability in PAEC monolayers [24], and mice with an endothelial-specific knockout of *Bmpr2* showed increased leukocyte recruitment and reduced barrier function [39]. In humans, aberrant immune regulation is a key feature in a significant proportion of patients with IPAH and associated with clinical outcomes, with a small subset of patients showing immunoglobin reactivity to BMPRII [40].

## Therapeutic strategies targeting BMP signalling in PAH

There are many ongoing efforts targeting BMP signalling for PAH treatment which have been reviewed recently [41–44]. Some focus on directly enhancing the cell surface BMPRII expression and such efforts include: (1) ataluren/PTC124 which promotes the read-through of stop-gain *BMPR2* mutations [45,46]; (2) small chemical chaperons such as 4-phenylbutyrate (4BA) which promote the secretion of misfolded BMPRII mutant proteins trapped in the endoplasmic reticulum [47]; (3) chloroquine and hydroxychloroquine which prevent lysosomal degradation of wild type BMPRII proteins [48]. However, these approaches are not specific to BMPRII, and the efficacy and potential side effects for treating PAH are yet to be seen in humans. Another approach employs low-dose FK506 which enhanced BMP signalling and reversed PAH in rodent models [49]. FK506 binds to FKBP12 and releases it from BMP type I receptors thereby enhancing BMP signalling. In a Phase IIa randomised placebo-controlled trial, FK506 was shown to be safe, increased *BMPR2* expression and improved 6-min walk distance in a subset of patients, but the overall efficacy is yet to be evaluated in a larger, multicentre trial [50]. Two approaches will be discussed further here: (1) targeting BMP9 signalling, and (2) Sotatercept, which has been approved by the FDA in March 2024 for treating PAH. Both approaches target the extracellular regulation of the TGF-β family signalling complex.

## Targeting BMP9 signalling in PAH and controversies over BMP9 signalling

Genetic and clinical evidence strongly supports that loss of BMP9 signalling contributes to the pathogenesis of PAH. Rare heterozygous detrimental mutations in *GDF2* (encoding BMP9) have been found in several large cohort genomic studies [51–53]. Patients with pathogenic BMP9 mutations have lower plasma levels of BMP9 and BMP10 [52,54]. Several homozygous null mutations in *GDF2* have also been identified in paediatric PAH patients and circulating BMP9 is unmeasurable in these patients [54–57].

BMP9 is secreted from the liver and circulates at active concentrations, acting constitutively on vascular endothelium as a vascular quiescence factor [27]. BMP9 and BMP10 are the only two known high affinity ligands for ALK1. They form a signalling complex with ALK1 and BMPRII in endothelial cells and signal potently with an EC_50_ below 0.1 ng/ml [30,58]. While BMPRII protein levels in endothelial cells reduce rapidly after protein synthesis inhibition [59], BMP9 induces *BMPR2* mRNA expression in endothelial cells [29], thus forming a dynamic balance. Based on the hypothesis that the loss of endothelial BMPRII and circulating BMP9 could be rescued by supplementation of BMP9, it was reported in 2015 that administration of recombinant BMP9 reversed PAH in three different rodent models: a genetic mouse knock-in model containing a human *BMPR2* mutation, the monocrotaline (MCT) — induced rat model, and a rat model induced by Sugen alongside chronic hypoxia (Sugen-Hypoxia) [22]. Here, BMP9 was also shown to confer protection against endothelial dysfunction. For example, *in vitro*, treatment of hPAECs with BMP9 offered protection against apoptosis induced by TNF-α and cycloheximide co-treatment, and BMP9 prevented excessive permeability in PAEC monolayer induced by TNF-α or LPS [22]. Of note, a potential beneficial role of BMP9 has also been reported in sepsis. Human patients with sepsis have lower BMP9 concentrations at admission, and lower BMP9 concentrations are associated with higher risk of death. BMP9 treatment improved the outcome in mice with experimental sepsis [60].

However, Tu et al. [61] reported in 2019 that BMP9 knockout mice, or mice administrated with a neutralising anti-BMP9 antibody, were significantly protected against chronic hypoxia-induced pulmonary hypertension. Furthermore, they showed that ALK1-Fc treatment rescued rat PAH models induced either by MCT or Sugen-Hypoxia. Such results are intriguing as they are different from hypotheses derived from human genetics. Further studies using BMP9 and BMP10 double knockout mice revealed even more complex picture where the double knockout mice developed high-output heart failure [62]. Here they also showed that BMP9 contributed to the hypoxia-induced pulmonary vascular remodelling, whereas BMP10 played a role in hypoxia-induced cardiac remodelling. In a separate study, BMP9 and BMP10 were shown to directly act on vascular smooth muscle cells and affect the contractility state of the SMCs [63]. Table 1 summarises *in vivo* studies supporting BMP9 agonist or antagonist approaches in the context of PAH.

**Table 1.** Summary of *in vivo* studies related to BMP9 agonist and antagonist approaches in PAH preclinical models

PMID	Journal, year	Title of paper, reference number	Key findings related to BMP9 agonist or antagonist approaches in PAH models
26076038	*Nat. Med.*, 2015	Selective enhancement of endothelial BMPR-II with BMP9 reverses pulmonary arterial hypertension [22]	Administration of recombinant BMP9 reversed established PAH in *Bmpr2* R899X knock-in mice, as well as in rat PAH models induced by monocrotaline or Sugen-Hypoxia.
30636542	*Circ. Res.*, 2019	Selective BMP-9 inhibition partially protects against experimental pulmonary hypertension [61]	;Bmp9^−/−^ mice and its inhibition in C57BL/6 mice using neutralising anti-BMP9 antibodies substantially prevent against chronic hypoxia-induced pulmonary hypertension. The BMP9/BMP10 ligand trap ALK1 ECD administered in monocrotaline or Sugen/Hypoxia (SuHx) rats substantially attenuates proliferation of pulmonary vascular cells, inflammatory cell infiltration, and regresses established pulmonary hypertension in rats.
30312106	*Am. J. Respir. Crit. Care Med.*, 2019	Bone morphogenetic protein 9 is a mechanistic biomarker of portopulmonary hypertension [64]	Administration of BMP9 ligand trap ALK1-Fc exacerbated pulmonary hypertension and pulmonary vascular remodelling in mice treated with hypoxia.
33334130	*Circulation*, 2021	BMP9 and BMP10 act directly on vascular smooth muscle cells for generation and maintenance of the contractile state [63]	BMP9 KO/BMP10 iKO in right atrium: dramatic changes in vascular tone and diminution of the VSMC layer with attenuated contractility and decreased systemic as well as right ventricular systolic pressure. Deletion of *Acvrl1* (encoding Alk1) in VSMCs recapitulated the Bmp9/10 phenotype in pulmonary but not in aortic and coronary arteries.
34086873	*Cardiovasc. Res.*, 2022	Different cardiovascular and pulmonary phenotypes for single- and double-knock-out mice deficient in BMP9 and BMP10 [62]	BMP9 contributes to chronic hypoxia-induced pulmonary vascular remodelling, whereas BMP10 plays a role in hypoxia-induced cardiac remodelling in mice. Combined deficiency in Bmp9 and Bmp10 led to vascular defects resulting in a decrease in peripheral vascular resistance and blood pressure and the progressive development of high-output heart failure and pulmonary hemosiderosis.

The controversial observations on BMP9 extend to *in vitro* cell biological studies. A recent report suggests that loss of endothelial *BMPR2* expression reverses the endothelial response to BMP9, causing enhanced proliferation [65]. It is difficult to compare this study with previous published data as it uses very different treatment conditions, i.e. 1 ng/ml of BMP9 treatment which is well above the concentrations measured in human plasma [54], and the experiments were performed in full growth media which already contain high concentrations of BMP9. Of note, most of the reports use serum-restricted conditions when evaluating BMP9 signalling. Altered BMP9 response in endothelial cells derived from PAH patients was also observed in another study. Here BMP9 induced unfavourable endothelial-to-mesenchymal transition only in endothelial cells isolated from PAH patients but not healthy controls [66].

With the current understanding of BMP9 and BMP10 signalling [67,68], it is difficult to reconcile these controversial observations. The functions of BMP9 and BMP10 may have more complexity than hitherto recognised, i.e. more cell types might be involved when investigating BMP9 and BMP10 effects *in vivo*. One might interpret such controversial findings as BMP9 exerts different roles at different stages of PAH; a beneficial role during the initial stage of PAH (where genetic studies are very powerful in identifying the underlying cause of the disease) and a more complicated role during the late stage of the disease which can often be demonstrated in cells isolated from patients who are at advanced stages of PAH. Such a scenario was seen for TGF-β, where it could act as either a tumour suppressor or a tumour promotor depending on different stages of the tumour development [69].

## Sotatercept and ActRIIA-mediated signalling

Sotatercept, a fusion protein comprising the extracellular ligand binding domain of ActRIIA fused to the Immunoglobin Fc domain, is approved by the FDA for treating Group 1 PAH and is the first PAH therapy targeting the TGF-β superfamily. In a Phase III clinical trial for PAH [70], Sotatercept improved the 6-min walk distance primary endpoint as well as in eight out of nine secondary efficacy endpoints compared with placebo controls, including time to death and clinical worsening [70,71]. Adverse events include epistaxis, telangiectasia, increased haemoglobin levels, thrombocytopenia and increased blood pressure, some of which may be related to the known affinity of ActRII-A with BMP and GDF ligands.

The mechanism of action of Sotatercept is still not fully understood. An early study suggests that it restores the balance between SMAD1/5/9 and SMAD2/3 signalling in PAH [72]. Here the authors showed that treatment with ActRIIA-Fc reversed elevated phospho-SMAD2/3 in a rat MCT model, but no restoration of reduced phospho-SMAD1/5/9 was observed in either rat MCT or rat Sugen-Hypoxia models [72]. Of note, Sotatercept is a ligand trap for activins and potentially also BMP9 and BMP10, so increased phospho-SMAD1/5/9 is not a direct outcome expected from Sotatercept treatment. Another study showed that treatment of ActRIIA-Fc in a Sugen-Hypoxia rat model normalised inflammatory response in the lungs, and importantly, the treatment suppressed the elevation of *Inhba* (encoding activin A, or ActA) and *Inhbb* (encoding ActB) expression in the right ventricle of Sugen-Hypoxia rats [73]. However, Sotatercept is an extracellular ligand trap and SMAD proteins are the intracellular mediators of signalling; these data still do not reveal the mode of action of Sotatercept at direct protein-protein interaction levels. We still do not know which target ligand or ligands are trapped by Sotatercept for its efficacy in PAH, nor do we know the major cell type that is responsible for the efficacy of Sotatercept.

**Figure 2.**
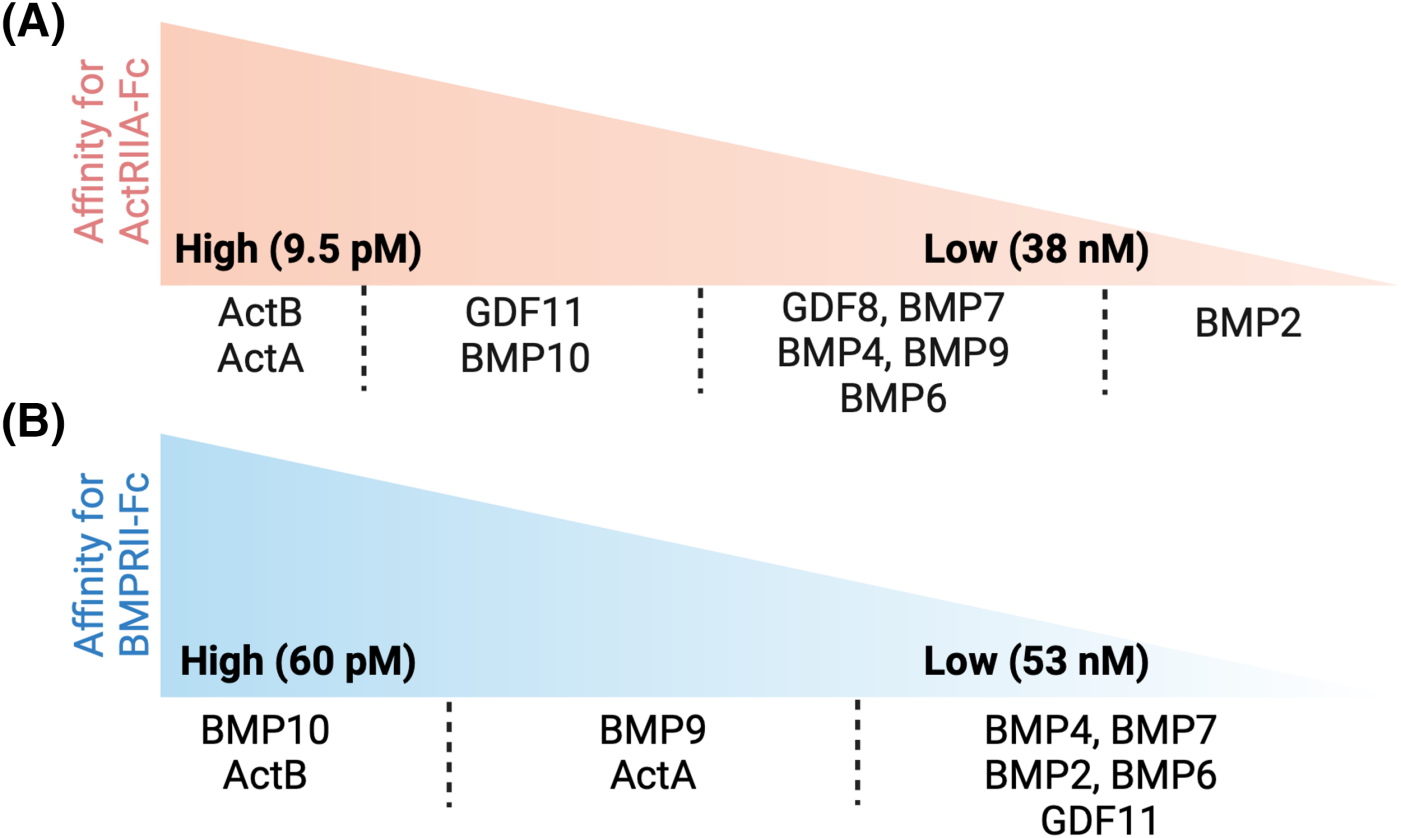
Summary of different ligand binding affinities for ActRIIA and BMPRII. Ligands with high to low affinities for ActRIIA-Fc (**A**) and BMPRII-Fc (**B**), based on the binding data in Table 2 and Table 3. *K_D_* values for the high and low affinities are taken from Table 2 and Table 3.

**Figure 3.**
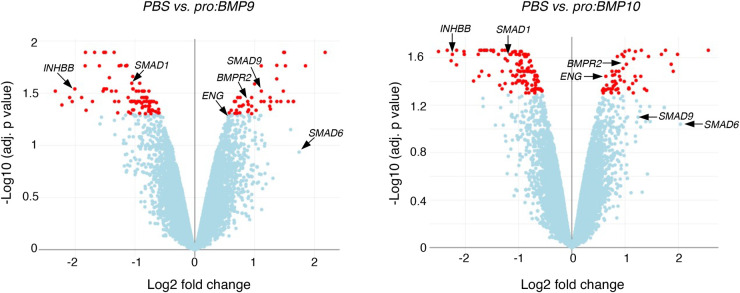
BMP9 and BMP10 suppress ActB expression in human pulmonary artery endothelial cells (hPAECs). Serum-starved hPAECs were treated with PBS, prodomain-bound BMP9 (pro:BMP9), or pro:BMP10 at concentrations equivalent to 0.4 ng/ml growth factor domain alone. After 5 hours, cells were harvested, and RNA was extracted for microarray analysis. Volcano plots showing the differential gene expression. Hits with adjusted P values less than 0.05 are shown in red. *INHBB*, which encoding ActB, is highlighted. Also highlighted are the components of BMP signalling (Figure 1) that are regulated by BMP9 and BMP10. Data for PBS vs pro:BMP9 has been published previously [30].

Ligands with high affinities for ActRIIA are more likely to be bound and inhibited by Sotatercept. ActRIIA and ActRIIB are the major type II receptors for Activins, and ActRIIA has been shown to mediate signals from multiple BMP ligands using siRNA approaches [29,34,74]. In Biacore direct binding assays, ActRIIA-Fc has been shown to bind multiple TGF-β family ligands with high affinities (Table 2). For activin ligands, ActA and ActB bind to ActRIIA-Fc with the highest affinity [77,79], whereas ActC only binds to ActRIIA transiently and no reported data on ActE. ActRIIA-Fc binds tightly to several GDF and BMP ligands, with *K_D_* in the sub-nanomolar range for GDF11 and BMP10, and in the nanomolar range for GDF8, BMP7, BMP4, BMP9 and BMP6 (Table 2, Figure 2). Interestingly, many of these ligands also bind BMPRII with high affinity (Table 3, Figure 2); for example, ActB binds to BMPRII-Fc with comparable affinity to BMP10 and stronger than many other BMP ligands [77,79]. ActA also binds BMPRII-Fc with very high affinity, but weaker than ActB or BMP10. Serum levels of both ActA and ActB are significantly elevated in PAH patients [84]. *INHBA* (encoding ActA) is highly expressed in lung microvascular endothelial cells [85]. PAECs isolated from the lungs of patients with IPAH synthesised more *INHBA* mRNA and released more ActA protein into the culture medium [85]. ActA has been shown to be capable of inhibiting BMP9 but not BMP2 and BMP4 signalling in two multiple myeloma cell lines [86], but such inhibition was not observed in endothelial cells [87]. Interestingly, it was suggested that BMPRII inhibits activin signalling via ALK2 because knocking down *BMPR2* by siRNA lead to enhanced ActA-phospho-SMAD1/5 signalling via ALK2 in multiple myeloma cells [88]. This observation agrees with another report that ActA forms a non-signalling complex with ALK2 and type II Activin/BMP receptors [89]. Taken together, these reports could potentially point to a hypothesis that elevated ActA and ActB contribute to the disease progression of PAH, partly by competitive binding to BMPRII thereby further reducing the availability of BMPRII for BMP signalling and exacerbating BMPRII loss. Another potential mechanism suggested by a recent report is that binding of ActA to BMPRII leads to endocytosis of BMPRII protein, hence further reducing the cell surface BMPRII [85]. However, increased BMPRII levels or BMPRII-mediated signalling after ActRIIA-Fc or Sotatercept treatment has not been reported in either preclinical or clinical data. Nevertheless, both hypotheses predict that Sotatercept should have a beneficial effect in PAH by directly sequestering the elevated ActA and ActB. Of interest, treatment of PAECs with either BMP9 or BMP10, both in the physiologically relevant prodomain-bound forms and at physiologically relevant concentrations, can supress the expression of the *INHBB* gene which encodes ActB (Figure 3) [90], in agreement with a beneficial effect of a BMP9 agonist approach for treating PAH.

**Table 2.** Affinities of different ligands for ActRIIA measured by surface plasmon resonance (Biacore)

Ligands	Dissociation constant (*K_D_*) and reference paper
ActA	22 pM [75], 23 pM [76], 43 pM [77]^a^, 59 pM [78]^b^, 90 pM [79]^a^
ActB	9.5 pM [75], 61 pM [77]^a^, 53 pM [79]^a^
ActC	Transient binding [75]
ActE	No report
GDF8	3.77 nM [77]^a^
GDF11	573 pM [77]^a^, 52 pM [79]^a^
BMP2	38 nM [78]^b^, 20.6 nM [80]^c^, ∼68 nM [81]^b^
BMP4	∼3 nM [82], 3.51 nM [77]^a^, 11.9 nM [80]^c^
BMP6	10.5 nM [77]^a^
BMP7	1.59 nM [77]^a^, 1.2 nM [78]^b^, No binding [79]^a^
BMP9	6.43 nM [83]^c^ (25°C)
BMP10	88.6 pM [83] (25°C), 381 pM [77], 1 nM [79]^a^

Data were obtained using human ActRIIA-Fc immobilised on the Biacore chip and titrating a range of ligand concentrations apart from those specified in the footnotes below. Data using immobilised ligands are not included here. Although it is difficult to directly compare the exact *K_D_* values from different studies, the reported values are in general agreement, and the values obtained from the same study under the same conditions are directly comparable.

a From single injection on Biacore.

b Used mouse ActRIIA, and not Fc fusion.

c By Steady-state analysis.

**Table 3.** Affinities of different ligands for BMPRII measured by surface plasmon resonance (Biacore)

Ligands	Dissociation constant (*K_D_*) and reference paper
ActA	9.0 nM [77]^a^, 9.6 nM [79]^a^
ActB	0.7 nM [79], 0.6 nM [77], 2.3 nM [79]^a^, 0.7 nM [79]
GDF11	53.3 nM [77]^a^
BMP2	41.4 [80]^b^
BMP4	7.4 nM [82], 26.3 nM [80]^b^
BMP6	44.5 nM [77]^a^
BMP7	39 nM [77]^a^, 13 nM [79]^a^
BMP9	7.4 nM [77]^a^, 0.6 nM [83]
BMP10	2.4 nM [79], 2.1 nM [79]^a^, 0.2 nM [77]^a^, 0.06 nM [83]

Data were obtained using human BMPRII-Fc immobilised on the Biacore chip and titrating a range of ligand concentrations apart from those specified in the footnotes below. Data using immobilised ligands are not included here. Although it is difficult to directly compare the exact *K_D_* values from different studies, the reported values are in general agreement, and the values obtained from the same study under the same conditions are directly comparable.

a From single injection on Biacore.

b By Steady-state analysis.

## Summary and future directions

Human genetics and pre-clinical studies both support a fundamental role of BMP signalling in the pathogenesis of PAH. BMP and activin signalling complexes are intertwined at multiple levels, involving the competitive binding of all three BMP type II receptors, BMPRII, ActRIIA and ActRIIB. The positive outcome from the Sotatercept Phase III trial strongly suggests that dysregulated BMP signalling in PAH also involves activin signalling. A more in-depth investigation of the effect of ActA and ActB in PAH models and patient samples is warranted. Another area of BMP signalling in PAH that is not covered in this review is the shared mutations of endothelial BMP signalling components in PAH and hereditary haemorrhagic telangiectasia, which will be another important and intriguing topic to review.

## Perspectives

- *The importance of the field*: pathogenic mutations in multiple components of BMP signalling pathways have been identified in human genetic studies on PAH, supporting a crucial role of dysregulated BMP signalling in the pathogenesis of PAH. Sotatercept is the first FDA-approved therapeutic modality that directly targets extracellular regulation of TGF-β/BMP signalling.
- *Summary of the current thinking*: compromised endothelial BMP signalling involving ALK1 and BMPRII is likely an initial trigger for PAH. Aberrant BMP signalling in other cell types and other TGF-β family ligands and receptors may also contribute to the pathogenesis of PAH.
- *Future directions*: a deeper mechanistic insight into the extracellular regulation of signalling from BMPs, activins and GDFs may provide novel therapeutic opportunities. This could be achieved by *in vitro* cell signalling assays in a physiologically relevant context, such as using human primary cells, patient cells and co-culture models, and coupled with biochemical and structural studies to address the direct protein–protein interactions amongst ligands and receptors.

## Open Access

Open access for this article was enabled by the participation of University of Cambridge in an all-inclusive *Read & Publish* agreement with Portland Press and the Biochemical Society under a transformative agreement with JISC.

## Abbreviations

ALK1: activin receptor-like kinase 1
BMP: bone morphogenetic protein
ECD: extracellular domain
GDF: growth and differentiation factor
hPAEC: human pulmonary artery endothelial cell
IPAH: idiopathic PAH
MCT: monocrotaline
PAH: pulmonary arterial hypertension
PASMC: pulmonary artery smooth muscle cells
TGF-β: transforming growth factor-β
TNF-α: tumour necrosis factor-α

## References

[1] Humbert, M., Sitbon, O., Chaouat, A., Bertocchi, M., Habib, G., Gressin, V. et al. (2006) Pulmonary arterial hypertension in France: results from a national registry. Am. J. Respir. Crit. Care Med. 173, 1023–1030 10.1164/rccm.200510-1668OC 16456139

[2] Peacock, A.J., Murphy, N.F., McMurray, J.J., Caballero, L. and Stewart, S. (2007) An epidemiological study of pulmonary arterial hypertension. Eur. Respir. J. 30, 104–109 10.1183/09031936.00092306 17360728

[3] Rabinovitch, M. (2012) Molecular pathogenesis of pulmonary arterial hypertension. J. Clin. Invest. 122, 4306–4313 10.1172/JCI60658 23202738 PMC3533531

[4] Lau, E.M.T., Giannoulatou, E., Celermajer, D.S. and Humbert, M. (2017) Epidemiology and treatment of pulmonary arterial hypertension. Nat. Rev. Cardiol. 14, 603–614 10.1038/nrcardio.2017.84 28593996

[5] Southgate, L., Machado, R.D., Graf, S. and Morrell, N.W. (2020) Molecular genetic framework underlying pulmonary arterial hypertension. Nat. Rev. Cardiol. 17, 85–95 10.1038/s41569-019-0242-x 31406341

[6] Eichstaedt, C.A., Belge, C., Chung, W.K., Graf, S., Grunig, E., Montani, D. et al. (2023) Genetic counselling and testing in pulmonary arterial hypertension: a consensus statement on behalf of the International Consortium for Genetic Studies in PAH. Eur. Respir. J. 61, 201471 10.1183/13993003.01471-2022 PMC9947314 36302552

[7] Evans, J.D., Girerd, B., Montani, D., Wang, X.J., Galie, N., Austin, E.D. et al. (2016) BMPR2 mutations and survival in pulmonary arterial hypertension: an individual participant data meta-analysis. Lancet Respir. Med. 4, 129–137 10.1016/S2213-2600(15)00544-5 26795434 PMC4737700

[8] Girerd, B., Montani, D., Jais, X., Eyries, M., Yaici, A., Sztrymf, B. et al. (2016) Genetic counselling in a national referral centre for pulmonary hypertension. Eur. Respir. J. 47, 541–552 10.1183/13993003.00717-2015 26699722

[9] Machado, R.D., Southgate, L., Eichstaedt, C.A., Aldred, M.A., Austin, E.D., Best, D.H. et al. (2015) Pulmonary arterial hypertension: a current perspective on established and emerging molecular genetic defects. Hum. Mutat. 36, 1113–1127 10.1002/humu.22904 26387786 PMC4822159

[10] Welch, C.L., Aldred, M.A., Balachandar, S., Dooijes, D., Eichstaedt, C.A., Graf, S. et al. (2023) Defining the clinical validity of genes reported to cause pulmonary arterial hypertension. Genet Med. 25, 100925 10.1016/j.gim.2023.100925 37422716 PMC10766870

[11] Li, W., Morrell, N.W. (2022) Endothelial bone morphogenetic protein signaling in pulmonary arterial hypertension. In Encyclopedia of Cell Biology, 2nd edn, 6, pp. 551–562, ELSEVIER, 2023. 10.1016/B978-0-12-821618-7.00246-7

[12] Larkin, E.K, Newman, J.H, Austin, E.D, Hemnes, A.R, Wheeler, L., Robbins, I.M. et al. Longitudinal analysis casts doubt on the presence of genetic anticipation in heritable pulmonary arterial hypertension. Am. J. Respir. Crit. Care Med. 2012;186:892–896 10.1164/rccm.201205-0886OC 22923661 PMC3530218

[13] Al Tabosh, T., Al Tarrass, M., Tourvieilhe, L., Guilhem, A., Dupuis-Girod, S. and Bailly, S. (2024) Hereditary hemorrhagic telangiectasia: from signaling insights to therapeutic advances. J. Clin. Invest. 134, e176379 10.1172/JCI176379 38357927 PMC10866657

[14] Miller, D., Hill, C.S. (2023) TGF-β family signaling. In Encyclopedia of Cell Biology, 2nd edn, pp. 46–61, Elsevier, Amsterdam

[15] Hinck, A.P., Mueller, T.D. and Springer, T.A. (2016) Structural biology and evolution of the TGF-beta family. Cold Spring Harb. Perspect. Biol. 8, a022103 10.1101/cshperspect.a022103 27638177 PMC5131774

[16] Ratcliff, M., Zhou, R.X., Jermutus, L. and Hyvonen, M. (2021) The role of pro-domains in human growth factors and cytokines. Biochem. Soc. Trans. 49, 1963–1973 10.1042/BST20200663 34495310 PMC8589418

[17] Martinez-Hackert, E., Sundan, A. and Holien, T. (2021) Receptor binding competition: a paradigm for regulating TGF-beta family action. Cytokine Growth Factor Rev. 57, 39–54 10.1016/j.cytogfr.2020.09.003 33087301 PMC7897244

[18] Grynblat, J., Bogaard, H.J., Eyries, M., Meyrignac, O., Savale, L., Jais, X. et al. (2024) Pulmonary vascular phenotype identified in patients with GDF2 (BMP9) or BMP10 variants: an international multicentre study. Eur. Respir. J. 63, 2301634 10.1183/13993003.01634-2023 38514094

[19] Budhiraja, R., Tuder, R.M. and Hassoun, P.M. (2004) Endothelial dysfunction in pulmonary hypertension. Circulation 109, 159–165 10.1161/01.CIR.0000102381.57477.50 14734504

[20] Diebold, I., Hennigs, J.K., Miyagawa, K., Li, C.G., Nickel, N.P., Kaschwich, M. et al. (2015) BMPR2 preserves mitochondrial function and DNA during reoxygenation to promote endothelial cell survival and reverse pulmonary hypertension. Cell Metab. 21, 596–608 10.1016/j.cmet.2015.03.010 25863249 PMC4394191

[21] Hopper, R.K., Moonen, J.R., Diebold, I., Cao, A., Rhodes, C.J., Tojais, N.F. et al. (2016) In pulmonary arterial hypertension, reduced BMPR2 promotes endothelial-to-mesenchymal transition via HMGA1 and its target slug. Circulation 133, 1783–1794 10.1161/CIRCULATIONAHA.115.020617 27045138 PMC4856565

[22] Long, L., Ormiston, M.L., Yang, X., Southwood, M., Graf, S., Machado, R.D. et al. (2015) Selective enhancement of endothelial BMPR-II with BMP9 reverses pulmonary arterial hypertension. Nat. Med. 21, 777–785 10.1038/nm.3877 26076038 PMC4496295

[23] Teichert-Kuliszewska, K., Kutryk, M.J., Kuliszewski, M.A., Karoubi, G., Courtman, D.W., Zucco, L. et al. (2006) Bone morphogenetic protein receptor-2 signaling promotes pulmonary arterial endothelial cell survival: implications for loss-of-function mutations in the pathogenesis of pulmonary hypertension. Circ. Res. 98, 209–217 10.1161/01.RES.0000200180.01710.e6 16357305

[24] Burton, V.J., Ciuclan, L.I., Holmes, A.M., Rodman, D.M., Walker, C. and Budd, D.C. (2011) Bone morphogenetic protein receptor II regulates pulmonary artery endothelial cell barrier function. Blood 117, 333–341 10.1182/blood-2010-05-285973 20724539

[25] Chowdhury, H.M., Sharmin, N., Yuzbasioglu Baran, M., Long, L., Morrell, N.W., Trembath, R.C. et al. (2019) BMPRII deficiency impairs apoptosis via the BMPRII-ALK1-BclX-mediated pathway in pulmonary arterial hypertension. Hum. Mol. Genet. 28, 2161–2173 10.1093/hmg/ddz047 30809644

[26] Hong, K.H., Lee, Y.J., Lee, E., Park, S.O., Han, C., Beppu, H. et al. (2008) Genetic ablation of the BMPR2 gene in pulmonary endothelium is sufficient to predispose to pulmonary arterial hypertension. Circulation 118, 722–730 10.1161/CIRCULATIONAHA.107.736801 18663089 PMC3920834

[27] Bidart, M., Ricard, N., Levet, S., Samson, M., Mallet, C., David, L. et al. (2012) BMP9 is produced by hepatocytes and circulates mainly in an active mature form complexed to its prodomain. Cell. Mol. Life Sci. 69, 313–324 10.1007/s00018-011-0751-1 21710321 PMC11114909

[28] Jiang, H., Salmon, R.M., Upton, P.D., Wei, Z., Lawera, A., Davenport, A.P. et al. (2016) The prodomain-bound form of bone morphogenetic protein 10 is biologically active on endothelial cells. J. Biol. Chem. 291, 2954–2966 10.1074/jbc.M115.683292 26631724 PMC4742757

[29] Upton, P.D., Davies, R.J., Trembath, R.C. and Morrell, N.W. (2009) Bone morphogenetic protein (BMP) and activin type II receptors balance BMP9 signals mediated by activin receptor-like kinase-1 in human pulmonary artery endothelial cells. J. Biol. Chem. 284, 15794–15804 10.1074/jbc.M109.002881 19366699 PMC2708876

[30] Li, W., Long, L., Yang, X., Tong, Z., Southwood, M., King, R. et al. (2021) Circulating BMP9 protects the pulmonary endothelium during inflammation-induced lung injury in mice. Am. J. Respir. Crit. Care Med. 203, 1419–1430 10.1164/rccm.202005-1761OC 33320799 PMC8456542

[31] Guo, J., Liu, B., Thorikay, M., Yu, M., Li, X., Tong, Z. et al. (2022) Crystal structures of BMPRII extracellular domain in binary and ternary receptor complexes with BMP10. Nat. Commun. 13, 2395 10.1038/s41467-022-30111-2 35504921 PMC9064986

[32] Yang, X., Long, L., Southwood, M., Rudarakanchana, N., Upton, P.D., Jeffery, T.K. et al. (2005) Dysfunctional Smad signaling contributes to abnormal smooth muscle cell proliferation in familial pulmonary arterial hypertension. Circ. Res. 96, 1053–1063 10.1161/01.RES.0000166926.54293.68 15845886

[33] Yang, J., Li, X., Li, Y., Southwood, M., Ye, L., Long, L. et al. (2013) Id proteins are critical downstream effectors of BMP signaling in human pulmonary arterial smooth muscle cells. Am. J. Physiol. Lung Cell. Mol. Physiol. 305, L312–L321 10.1152/ajplung.00054.2013 23771884 PMC3891012

[34] Yu, P.B., Beppu, H., Kawai, N., Li, E. and Bloch, K.D. (2005) Bone morphogenetic protein (BMP) type II receptor deletion reveals BMP ligand-specific gain of signaling in pulmonary artery smooth muscle cells. J. Biol. Chem. 280, 24443–24450 10.1074/jbc.M502825200 15883158

[35] Hurst, L.A., Dunmore, B.J., Long, L., Crosby, A., Al-Lamki, R., Deighton, J. et al. (2017) TNFalpha drives pulmonary arterial hypertension by suppressing the BMP type-II receptor and altering NOTCH signalling. Nat. Commun. 8, 14079 10.1038/ncomms14079 28084316 PMC5241886

[36] Soon, E., Crosby, A., Southwood, M., Yang, P., Tajsic, T., Toshner, M. et al. (2015) Bone morphogenetic protein receptor type II deficiency and increased inflammatory cytokine production. A gateway to pulmonary arterial hypertension. Am. J. Respir. Crit. Care Med. 192, 859–872 10.1164/rccm.201408-1509OC 26073741 PMC4613895

[37] Davies, R.J., Holmes, A.M., Deighton, J., Long, L., Yang, X., Barker, L. et al. (2012) BMP type II receptor deficiency confers resistance to growth inhibition by TGF-beta in pulmonary artery smooth muscle cells: role of proinflammatory cytokines. Am. J. Physiol. Lung Cell. Mol. Physiol. 302, L604–L615 10.1152/ajplung.00309.2011 22227206 PMC3311534

[38] Pickworth, J., Rothman, A., Iremonger, J., Casbolt, H., Hopkinson, K., Hickey, P.M. et al. (2017) Differential IL-1 signaling induced by BMPR2 deficiency drives pulmonary vascular remodeling. Pulm. Circ. 7, 768–776 10.1177/2045893217729096 28828907 PMC5703124

[39] Burton, V.J., Holmes, A.M., Ciuclan, L.I., Robinson, A., Roger, J.S., Jarai, G. et al. (2011) Attenuation of leukocyte recruitment via CXCR1/2 inhibition stops the progression of PAH in mice with genetic ablation of endothelial BMPR-II. Blood 118, 4750–4758 10.1182/blood-2011-05-347393 21900197 PMC3208288

[40] Jones, R.J., De Bie, E., Groves, E., Zalewska, K.I., Swietlik, E.M., Treacy, C.M. et al. (2022) Autoimmunity is a significant feature of idiopathic pulmonary arterial hypertension. Am. J. Respir. Crit. Care Med. 206, 81–93 10.1164/rccm.202108-1919OC 35316153 PMC7613913

[41] Sharmin, N., Nganwuchu, C.C. and Nasim, M.T. (2021) Targeting the TGF-beta signaling pathway for resolution of pulmonary arterial hypertension. Trends Pharmacol. Sci. 42, 510–513 10.1016/j.tips.2021.04.002 33966900

[42] Dunmore, B.J., Jones, R.J., Toshner, M.R., Upton, P.D. and Morrell, N.W. (2021) Approaches to treat pulmonary arterial hypertension by targeting BMPR2: from cell membrane to nucleus. Cardiovasc. Res. 117, 2309–2325 10.1093/cvr/cvaa350 33399862 PMC8479804

[43] Ali, M.K., Ichimura, K. and Spiekerkoetter, E. (2021) Promising therapeutic approaches in pulmonary arterial hypertension. Curr. Opin. Pharmacol. 59, 127–139 10.1016/j.coph.2021.05.003 34217109

[44] Dannewitz Prosseda, S., Ali, M.K. and Spiekerkoetter, E. (2020) Novel advances in modifying BMPR2 signaling in PAH. Genes (Basel) 12, 8 10.3390/genes12010008 33374819 PMC7824173

[45] Drake, K.M., Dunmore, B.J., McNelly, L.N., Morrell, N.W. and Aldred, M.A. (2013) Correction of nonsense BMPR2 and SMAD9 mutations by ataluren in pulmonary arterial hypertension. Am. J. Respir. Cell Mol. Biol. 49, 403–409 10.1165/rcmb.2013-0100OC 23590310 PMC3824059

[46] Long, L., Yang, X., Southwood, M., Moore, S., Crosby, A., Upton, P.D. et al. (2020) Targeting translational read-through of premature termination mutations in BMPR2 with PTC124 for pulmonary arterial hypertension. Pulm. Circ. 10, 2045894020935783 10.1177/2045894020935783 32733669 PMC7372630

[47] Dunmore, B.J., Yang, X., Crosby, A., Moore, S., Long, L., Huang, C. et al. (2020) 4PBA restores signaling of a cysteine-substituted mutant BMPR2 receptor found in patients with pulmonary arterial hypertension. Am. J. Respir. Cell Mol. Biol. 63, 160–171 10.1165/rcmb.2019-0321OC 32255665

[48] Long, L., Yang, X., Southwood, M., Lu, J., Marciniak, S.J., Dunmore, B.J. et al. (2013) Chloroquine prevents progression of experimental pulmonary hypertension via inhibition of autophagy and lysosomal bone morphogenetic protein type II receptor degradation. Circ. Res. 112, 1159–1170 10.1161/CIRCRESAHA.111.300483 23446737

[49] Spiekerkoetter, E., Tian, X., Cai, J., Hopper, R.K., Sudheendra, D., Li, C.G. et al. (2013) FK506 activates BMPR2, rescues endothelial dysfunction, and reverses pulmonary hypertension. J. Clin. Invest. 123, 3600–3613 10.1172/JCI65592 23867624 PMC3726153

[50] Spiekerkoetter, E., Sung, Y.K., Sudheendra, D., Scott, V., Del Rosario, P., Bill, M. et al. (2017) Randomised placebo-controlled safety and tolerability trial of FK506 (tacrolimus) for pulmonary arterial hypertension. Eur. Respir. J. 50, 1602449 10.1183/13993003.02449-2016 28893866

[51] Graf, S., Haimel, M., Bleda, M., Hadinnapola, C., Southgate, L., Li, W. et al. (2018) Identification of rare sequence variation underlying heritable pulmonary arterial hypertension. Nat. Commun. 9, 1416 10.1038/s41467-018-03672-4 29650961 PMC5897357

[52] Wang, X.J., Lian, T.Y., Jiang, X., Liu, S.F., Li, S.Q., Jiang, R. et al. (2019) Germline BMP9 mutation causes idiopathic pulmonary arterial hypertension. Eur. Respir. J. 53, 1801609 10.1183/13993003.01609-2018 30578397

[53] Eyries, M., Montani, D., Girerd, B., Favrolt, N., Riou, M., Faivre, L. et al. (2020) Familial pulmonary arterial hypertension by KDR heterozygous loss of function. Eur. Respir. J. 55, 1902165 10.1183/13993003.02165-2019 31980491

[54] Hodgson, J., Ruiz-Llorente, L., McDonald, J., Quarrell, O., Ugonna, K., Bentham, J. et al. (2021) Homozygous GDF2 nonsense mutations result in a loss of circulating BMP9 and BMP10 and are associated with either PAH or an “HHT-like” syndrome in children. Mol. Genet. Genomic Med. 9, e1685 10.1002/mgg3.1685 33834622 PMC8683697

[55] Upton, P., Richards, S., Bates, A., Niederhoffer, K.Y., Morrell, N.W. and Christian, S. (2023) A rare homozygous missense GDF2 (BMP9) mutation causing PAH in siblings: does BMP10 status contribute? Am. J. Med. Genet. A 191, 228–233 10.1002/ajmg.a.62996 36259599 PMC10092753

[56] Chomette, L., Hupkens, E., Romitti, M., Dewachter, L., Vachiery, J.L., Bailly, S. et al. (2023) Pediatric pulmonary arterial hypertension due to a novel homozygous GDF2 missense variant affecting BMP9 processing and activity. Am. J. Med. Genet. A 191, 2064–2073 10.1002/ajmg.a.63236 37249087

[57] Wang, G., Fan, R., Ji, R., Zou, W., Penny, D.J., Varghese, N.P. et al. (2016) Novel homozygous BMP9 nonsense mutation causes pulmonary arterial hypertension: a case report. BMC Pulm. Med. 16, 17 10.1186/s12890-016-0183-7 26801773 PMC4722683

[58] David, L., Mallet, C., Keramidas, M., Lamande, N., Gasc, J.M., Dupuis-Girod, S. et al. (2008) Bone morphogenetic protein-9 is a circulating vascular quiescence factor. Circ. Res. 102, 914–922 10.1161/CIRCRESAHA.107.165530 18309101 PMC2561062

[59] Dunmore, B.J., Drake, K.M., Upton, P.D., Toshner, M.R., Aldred, M.A. and Morrell, N.W. (2013) The lysosomal inhibitor, chloroquine, increases cell surface BMPR-II levels and restores BMP9 signalling in endothelial cells harbouring BMPR-II mutations. Hum. Mol. Genet. 22, 3667–3679 10.1093/hmg/ddt216 23669347 PMC3749859

[60] Bai, H., Lu, Q., Wu, C., Xu, F., Liu, J., Wang, K. et al. (2024) Bone morphogenetic protein 9 is a candidate prognostic biomarker and host-directed therapy target for sepsis. Sci. Transl. Med. 16, eadi3275 10.1126/scitranslmed.adi3275 38295185

[61] Tu, L., Desroches-Castan, A., Mallet, C., Guyon, L., Cumont, A., Phan, C. et al. (2019) Selective BMP-9 inhibition partially protects against experimental pulmonary hypertension. Circ. Res. 124, 846–855 10.1161/CIRCRESAHA.118.313356 30636542

[62] Bouvard, C., Tu, L., Rossi, M., Desroches-Castan, A., Berrebeh, N., Helfer, E. et al. (2022) Different cardiovascular and pulmonary phenotypes for single- and double-knock-out mice deficient in BMP9 and BMP10. Cardiovasc. Res. 118, 1805–1820 10.1093/cvr/cvab187 34086873 PMC9215199

[63] Wang, L., Rice, M., Swist, S., Kubin, T., Wu, F., Wang, S. et al. (2021) BMP9 and BMP10 act directly on vascular smooth muscle cells for generation and maintenance of the contractile state. Circulation 143, 1394–1410 10.1161/CIRCULATIONAHA.120.047375 33334130

[64] Nikolic, I., Yung, L.M., Yang, P., Malhotra, R., Paskin-Flerlage, S.D., Dinter, T. et al. (2019) Bone morphogenetic protein 9 is a mechanistic biomarker of portopulmonary hypertension. Am. J. Respir. Crit. Care Med. 199, 891–902 10.1164/rccm.201807-1236OC 30312106 PMC6444661

[65] Theilmann, A.L., Hawke, L.G., Hilton, L.R., Whitford, M.K.M., Cole, D.V., Mackeil, J.L. et al. (2020) Endothelial BMPR2 loss drives a proliferative response to BMP (bone morphogenetic protein) 9 via prolonged canonical signaling. Arterioscler. Thromb. Vasc. Biol. 40, 2605–2618 10.1161/ATVBAHA.119.313357 32998516 PMC7571847

[66] Szulcek, R., Sanchez-Duffhues, G., Rol, N., Pan, X., Tsonaka, R., Dickhoff, C. et al. (2020) Exacerbated inflammatory signaling underlies aberrant response to BMP9 in pulmonary arterial hypertension lung endothelial cells. Angiogenesis 23, 699–714 10.1007/s10456-020-09741-x 32813135 PMC7524846

[67] Upton, P.D., Dunmore, B.J., Li, W. and Morrell, N.W. (2022) An emerging class of new therapeutics targeting TGF, Activin, and BMP ligands in pulmonary arterial hypertension. Dev. Dyn. 252, 327–342 10.1002/dvdy.478 35434863 PMC10952790

[68] Desroches-Castan, A., Tillet, E., Bouvard, C. and Bailly, S. (2022) BMP9 and BMP10: two close vascular quiescence partners that stand out. Dev. Dyn. 251, 178–197 10.1002/dvdy.395 34240497

[69] Massague, J. (2008) TGFbeta in cancer. Cell 134, 215–230 10.1016/j.cell.2008.07.001 18662538 PMC3512574

[70] Hoeper, M.M., Badesch, D.B., Ghofrani, H.A., Gibbs, J.S.R., Gomberg-Maitland, M., McLaughlin, V.V. et al. (2023) Phase 3 trial of sotatercept for treatment of pulmonary arterial hypertension. N. Engl. J. Med. 388, 1478–1490 10.1056/NEJMoa2213558 36877098

[71] Humbert, M. (2023) Viewpoint: activin signalling inhibitors for the treatment of pulmonary arterial hypertension. Eur. Respir. J. 62, 2301726 10.1183/13993003.01726-2023 37918877

[72] Yung, L.M., Yang, P., Joshi, S., Augur, Z.M., Kim, S.S.J., Bocobo, G.A. et al. (2020) ACTRIIA-Fc rebalances activin/GDF versus BMP signaling in pulmonary hypertension. Sci. Transl. Med. 12, eaaz5660 10.1126/scitranslmed.aaz5660 32404506 PMC8259900

[73] Joshi, S.R., Liu, J., Bloom, T., Karaca Atabay, E., Kuo, T.H., Lee, M. et al. (2022) Sotatercept analog suppresses inflammation to reverse experimental pulmonary arterial hypertension. Sci. Rep. 12, 7803 10.1038/s41598-022-11435-x 35551212 PMC9098455

[74] David, L., Mallet, C., Mazerbourg, S., Feige, J.J. and Bailly, S. (2007) Identification of BMP9 and BMP10 as functional activators of the orphan activin receptor-like kinase 1 (ALK1) in endothelial cells. Blood 109, 1953–1961 10.1182/blood-2006-07-034124 17068149

[75] Goebel, E.J., Ongaro, L., Kappes, E.C., Vestal, K., Belcheva, E., Castonguay, R. et al. (2022) The orphan ligand, activin C, signals through activin receptor-like kinase 7. Elife 11, e78197 10.7554/eLife.78197 35736809 PMC9224996

[76] Aykul, S., Ni, W., Mutatu, W. and Martinez-Hackert, E. (2015) Human Cerberus prevents nodal-receptor binding, inhibits nodal signaling, and suppresses nodal-mediated phenotypes. PLoS One 10, e0114954 10.1371/journal.pone.0114954 25603319 PMC4300205

[77] Aykul, S. and Martinez-Hackert, E. (2016) Transforming growth factor-beta family ligands can function as antagonists by competing for type II receptor binding. J. Biol. Chem. 291, 10792–10804 10.1074/jbc.M115.713487 26961869 PMC4865925

[78] Greenwald, J., Groppe, J., Gray, P., Wiater, E., Kwiatkowski, W., Vale, W. et al. (2003) The BMP7/ActRII extracellular domain complex provides new insights into the cooperative nature of receptor assembly. Mol. Cell 11, 605–617 10.1016/s1097-2765(03)00094-7 12667445

[79] Chu, K.Y., Malik, A., Thamilselvan, V. and Martinez-Hackert, E. (2022) Type II BMP and activin receptors BMPR2 and ACVR2A share a conserved mode of growth factor recognition. J. Biol. Chem. 298, 102076 10.1016/j.jbc.2022.102076 35643319 PMC9234707

[80] Gipson, G.R., Nolan, K., Kattamuri, C., Kenny, A.P., Agricola, Z., Edwards, N.A. et al. (2023) Formation and characterization of BMP2/GDF5 and BMP4/GDF5 heterodimers. BMC Biol. 21, 16 10.1186/s12915-023-01522-4 36726183 PMC9893541

[81] Allendorph, G.P., Vale, W.W. and Choe, S. (2006) Structure of the ternary signaling complex of a TGF-beta superfamily member. Proc. Natl Acad. Sci. U.S.A. 103, 7643–7648 10.1073/pnas.0602558103 16672363 PMC1456805

[82] Aykul, S. and Martinez-Hackert, E. (2016) Determination of half-maximal inhibitory concentration using biosensor-based protein interaction analysis. Anal. Biochem. 508, 97–103 10.1016/j.ab.2016.06.025 27365221 PMC4955526

[83] Townson, S.A., Martinez-Hackert, E., Greppi, C., Lowden, P., Sako, D., Liu, J. et al. (2012) Specificity and structure of a high affinity activin receptor-like kinase 1 (ALK1) signaling complex. J. Biol. Chem. 287, 27313–27325 10.1074/jbc.M112.377960 22718755 PMC3431715

[84] Guignabert, C., Savale, L., Boucly, A., Thuillet, R., Tu, L., Ottaviani, M. et al. (2023) Serum and pulmonary expression profiles of the activin signaling system in pulmonary arterial hypertension. Circulation 147, 1809–1822 10.1161/CIRCULATIONAHA.122.061501 37096577

[85] Ryanto, G.R.T., Ikeda, K., Miyagawa, K., Tu, L., Guignabert, C., Humbert, M. et al. (2021) An endothelial activin A-bone morphogenetic protein receptor type 2 link is overdriven in pulmonary hypertension. Nat. Commun. 12, 1720 10.1038/s41467-021-21961-3 33741934 PMC7979873

[86] Olsen, O.E., Wader, K.F., Hella, H., Mylin, A.K., Turesson, I., Nesthus, I. et al. (2015) Activin A inhibits BMP-signaling by binding ACVR2A and ACVR2B. Cell Commun. Signal. 13, 27 10.1186/s12964-015-0104-z 26047946 PMC4467681

[87] Wood, J.H., Guo, J., Morrell, N.W. and Li, W. (2019) Advances in the molecular regulation of endothelial BMP9 signalling complexes and implications for cardiovascular disease. Biochem. Soc. Trans. 47, 779–791 10.1042/BST20180137 31127068

[88] Olsen, O.E., Sankar, M., Elsaadi, S., Hella, H., Buene, G., Darvekar, S.R. et al. (2018) BMPR2 inhibits activin and BMP signaling via wild-type ALK2. J. Cell Sci. 131, jcs213512 10.1242/jcs.213512 29739878

[89] Aykul, S., Corpina, R.A., Goebel, E.J., Cunanan, C.J., Dimitriou, A., Kim, H.J. et al. (2020) Activin A forms a non-signaling complex with ACVR1 and type II Activin/BMP receptors via its finger 2 tip loop. Elife 9, e54582 10.7554/eLife.54582 32515349 PMC7326492

[90] Lawera, A., Tong, Z., Thorikay, M., Redgrave, R.E., Cai, J., van Dinther, M. et al. (2019) Role of soluble endoglin in BMP9 signaling. Proc. Natl Acad. Sci. U.S.A. 116, 17800–17808 10.1073/pnas.1816661116 31431534 PMC6731690

